# Supplementary material for: Smartphone dependence, addiction, and insomnia among medical students during the COVID-19 pandemic
Source: PLoS One. 2025 Jul 31;20(7):e0329495. doi: 10.1371/journal.pone.0329495 (PMC12312953; doi:10.1371/journal.pone.0329495)
Supplement: Attachment — Submitted filename: Response to Editor.pdf [file pone.0329495.s004.pdf]

Editor's comments

1. Please complete all items on the Human Participants Research Checklist that are relevant for your submission, by following this link: http://journals.plos.org/plosone/s/file?id=dc11/PLOSOne_Human_Subjects_Research_Checklist.docx (Contact us at plosone@plos.org if you cannot access the document.) There may be overlap between the checklist items and other queries listed below; please address any duplicated queries both in your response email and on the checklist itself. Upload the completed Human Participants Research Checklist as file type "Other" when you re-submit your manuscript. This document is for internal journal use only and will not be published if your article is accepted. The requested information will help us to assess whether your submission complies with PLOS ONE's policies and adheres to applicable reporting standards. Note that your manuscript may be rejected if you provide incomplete or inadequate responses to the checklist questions and that changing the 'Section/Category' of your article does not affect this requirement.

   Our response: Thank you for your comments and for pointing out the need to complete the Human Participants Research Checklist. We have downloaded the document using the link provided and have completed all items relevant to our research.

   The completed checklist has been uploaded as an "Other" file into the resubmission system as indicated in the instructions. Please do not hesitate to get in touch if you need additional information or if there is anything that requires further clarification.

2. Please provide additional details regarding participant consent. In the ethics statement in the Methods and online submission information, please ensure that you have specified what type you obtained (for instance, written or verbal, and if verbal, how it was documented and witnessed). If your study included minors, state whether you obtained consent from parents or guardians. If the need for consent was waived by the ethics committee, please include this information.

   Our response: Thank you for your comment regarding the participant consent section. We have updated the manuscript to include the additional details requested: we specified that the consent obtained was informed and in writing for all participants, and that in the case of minors, consent was also obtained from their parents or legal guardians. We also reinforced the description of the ethical measures implemented, highlighting compliance with the principles of the Declaration of Helsinki and actions to ensure the confidentiality and well-being of the participants. The revised manuscript already includes these adjustments in the corresponding section; we remain attentive to any additional comments.

If you have any comments or recommendations, we are ready to respond.

Sincerely,

Mario J. Valladares-Garrido

Universidad Continental, Lima 15046, Peru; mvalladares@continental.edu.pe
