# Supplementary material for: Smartphone dependence, addiction, and insomnia among medical students during the COVID-19 pandemic
Source: PLoS One. 2025 Jul 31;20(7):e0329495. doi: 10.1371/journal.pone.0329495 (PMC12312953; doi:10.1371/journal.pone.0329495)
Supplement: Attachment — Submitted filename: PONE-D-24-56765_reviewer1.pdf [file pone.0329495.s003.pdf]

PLOS ONE

Smartphone dependence, addiction, and insomnia among medical students during the COVID-19 pandemic

--Manuscript Draft--

**Manuscript Number:**	PONE-D-24-56765
**Article Type:**	Research Article
**Full Title:**	Smartphone dependence, addiction, and insomnia among medical students during the COVID-19 pandemic
**Short Title:**	Smartphone dependence, addiction, and insomnia among medical students
**Corresponding Author:**	Mario J. Valladares-Garrido<br>Universidad Continental - Los Olivos Lima<br>PERU
**Keywords:**	smartphone; Insomnia; dependence; addiction; medical students
**Abstract:**	Introduction<br><br>During the COVID-19 pandemic, smartphone use among students increased, even before going to sleep, which resulted in an increased risk of dependence, addiction, and sleep problems such as insomnia and daytime somnolence. The objective of this study was to determine the association between smartphone dependence and addiction and insomnia among medical students.<br><br>Materials and methods<br><br>This was a secondary cross-sectional study of a cohort of medical students from Piura (Peru) during the COVID-19 pandemic in 2020. The study included 370 participants. Insomnia was assessed using the Insomnia Severity Index, and the extent of smartphone use was determined using the Smartphone Dependence and Addiction Scale. A chi-squared test was used for the bivariate analysis, and prevalence ratios (PR) were estimated using Poisson regression with crude and adjusted models and 95% confidence intervals (CI).<br><br>Results<br><br>Insomnia was reported by 67.8% of the participants. Smartphone dependence was 67.8%, while users with behaviors indicative of addiction represented 10.8%. The prevalence of insomnia was higher among students aged ≥26 years (80.0%), with symptoms of depression (79.3%) and anxiety (81.9%). Participants with smartphone dependence were 1.9 times more likely to develop insomnia than those without dependence (PR: 1.9; 95% CI: 1.42–2.54; p < 0.001).<br><br>Conclusion<br><br>Medical students experienced high levels of smartphone dependence, which were associated with a higher likelihood of developing insomnia.
**Order of Authors:**	Virgilio E. Failoc-Rojas
	Alicia Torres-Mera
	Darwin A. León-Figueroa
	David Lira
	Flor M. Santander-Hernández
	Miguel A. Guevara-Morales
	Cristian Díaz-Vélez
	Mario J. Valladares-Garrido

Powered by Editorial Manager® and ProduXion Manager® from Aries Systems Corporation

Additional Information:	
**Question**	**Response**
**Financial Disclosure**<br><br>Enter a financial disclosure statement that describes the sources of funding for the work included in this submission. Review the submission guidelines for detailed requirements. View published research articles from *PLOS ONE* for specific examples.<br><br>This statement is required for submission and **will appear in the published article** if the submission is accepted. Please make sure it is accurate.<br><br>**Funded studies**<br>Enter a statement with the following details:<br>• Initials of the authors who received each award<br>• Grant numbers awarded to each author<br>• The full name of each funder<br>• URL of each funder website<br>• Did the sponsors or funders play any role in the study design, data collection and analysis, decision to publish, or preparation of the manuscript?<br><br>Did you receive funding for this work?	The author(s) received no specific funding for this work.
**Competing Interests**<br><br>Use the instructions below to enter a competing interest statement for this submission. On behalf of all authors, disclose any competing interests that could be perceived to bias this work—acknowledging all financial support and any other relevant financial or non-financial competing interests.<br><br>This statement is required for submission and **will appear in the published article** if the submission is accepted. Please make sure it is accurate and that any funding sources listed in your Funding Information	The authors have declared that no competing interests exist.

later in the submission form are also declared in your Financial Disclosure statement.<br><br>View published research articles from *PLOS ONE* for specific examples.<br><br>**NO authors have competing interests**<br><br>Enter: *The authors have declared that no competing interests exist.*<br><br>**Authors with competing interests**<br><br>Enter competing interest details beginning with this statement:<br><br>*I have read the journal's policy and the authors of this manuscript have the following competing interests: [insert competing interests here]*<br><br>* typeset	
**Ethics Statement**<br><br>Enter an ethics statement for this submission. This statement is required if the study involved:<br><br>• Human participants<br>• Human specimens or tissue<br>• Vertebrate animals or cephalopods<br>• Vertebrate embryos or tissues<br>• Field research<br><br>Write "N/A" if the submission does not require an ethics statement.<br><br>General guidance is provided below. Consult the submission guidelines for detailed instructions. **Make sure that all information entered here is included in the Methods section of the manuscript.**	This study was approved by the Research Ethics Committee of César Vallejo University, Piura, Peru (102P-CEI-EPM-UCV-2020).

**Format for specific study types**<br><br>**Human Subject Research (involving human participants and/or tissue)**<br>• Give the name of the institutional review board or ethics committee that approved the study<br>• Include the approval number and/or a statement indicating approval of this research<br>• Indicate the form of consent obtained (written/oral) or the reason that consent was not obtained (e.g. the data were analyzed anonymously)<br><br>**Animal Research (involving vertebrate animals, embryos or tissues)**<br>• Provide the name of the Institutional Animal Care and Use Committee (IACUC) or other relevant ethics board that reviewed the study protocol, and indicate whether they approved this research or granted a formal waiver of ethical approval<br>• Include an approval number if one was obtained<br>• If the study involved *non-human primates*, add *additional details* about animal welfare and steps taken to ameliorate suffering<br>• If anesthesia, euthanasia, or any kind of animal sacrifice is part of the study, include briefly which substances and/or methods were applied<br><br>**Field Research**<br><br>Include the following details if this study involves the collection of plant, animal, or other materials from a natural setting:<br>• Field permit number<br>• Name of the institution or relevant body that granted permission	
**Data Availability**<br><br>Authors are required to make all data underlying the findings described fully available, without restriction, and from the time of publication. PLOS allows rare exceptions to address legal and ethical concerns. See the PLOS Data Policy and FAQ for detailed information.	No - some restrictions will apply

A Data Availability Statement describing where the data can be found is required at submission. Your answers to this question constitute the Data Availability Statement and **will be published in the article**, if accepted.<br><br>**Important:** Stating 'data available on request from the author' is not sufficient. If your data are only available upon request, select 'No' for the first question and explain your exceptional situation in the text box.<br><br>Do the authors confirm that all data underlying the findings described in their manuscript are fully available without restriction?	
**Describe where the data may be found in full sentences. If you are copying our sample text, replace any instances of XXX with the appropriate details.**<br><br>• If the data are **held or will be held in a public repository**, include URLs, accession numbers or DOIs. If this information will only be available after acceptance, indicate this by ticking the box below. For example: *All XXX files are available from the XXX database (accession number(s) XXX, XXX.).*<br>• If the data are all contained **within the manuscript and/or Supporting Information files**, enter the following: *All relevant data are within the manuscript and its Supporting Information files.*<br>• If neither of these applies but you are able to provide **details of access elsewhere**, with or without limitations, please do so. For example:<br><br>*Data cannot be shared publicly because of [XXX]. Data are available from the XXX Institutional Data Access / Ethics Committee (contact via XXX) for researchers who meet the criteria for access to confidential data.*<br><br>*The data underlying the results presented in the study are available from (include the name of the third party*	All relevant data are within the manuscript and its Supporting Information files.

and contact information or URL).<br>• This text is appropriate if the data are owned by a third party and authors do not have permission to share the data.<br><br>* typeset	
Additional data availability information:	

Powered by Editorial Manager® and ProduXion Manager® from Aries Systems Corporation

Manuscript Click here to access/download;Manuscript;Manuscript.docx

Original article

Smartphone dependence, addiction, and insomnia among medical students during the COVID-19 pandemic

Virgilio E. Failoc-Rojas [1,2], Alicia Torres-Mera [3], Darwin A. León-Figueroa [4], David Lira [5], Flor M. Santander-Hernández [6], Miguel A. Guevara-Morales [6], Cristian Díaz-Vélez [7], Mario J. Valladares-Garrido [8,9,10]*

[1] Vicerrectorado de Investigación, Universidad San Ignacio de Loyola, Lima, Peru

[2] Análisis estadísticos, MedStat-Educación e Investigación, Lima, Peru; virgiliofr@gmail.com (V.E.F.R)

[3] Facultad de Medicina, Universidad Nacional Pedro Ruiz Gallo, Lambayeque, Peru; rtorresme@unprg.edu.pe (A.T.M)

[4] Facultad de Medicina, Universidad de San Martin de Porres, Chiclayo, Peru; darwin_leon@usmp.pe (D.A.L.F)

[5] Servicio de Neurología, Instituto Peruano de Neurociencias, Lima, Perú; dlira@ipn.pe (D.L)

[6] Universidad Cesar Vallejo, Piura, Peru; santherzmedic@gmail.com (F.M.S.H), and maguevaram@ucvvirtual.edu.pe (M.A.G.M)

[7] Universidad Cesar Vallejo, Trujillo, Peru; cristiandiazv@hotmail.com (C.D.V)

[8] Universidad Continental, Lima, Peru; mvalladares@continental.edu.pe (M.V.G)

[9] Oficina de Inteligencia Sanitaria, Red Prestacional EsSalud Lambayeque, Chiclayo, Peru.

[10] Oficina de Epidemiología, Hospital Regional Lambayeque, Chiclayo, Peru.

* **Correspondence**: mvalladares@continental.edu.pe ; Tel.: +51 944 655 396.

Abstract

Introduction: During the COVID-19 pandemic, smartphone use among students increased, even before going to sleep, which resulted in an increased risk of dependence, addiction, and sleep problems such as insomnia and daytime somnolence. The objective of this study was to determine the association between smartphone dependence and addiction and insomnia among medical students.

Materials and methods: This was a secondary cross-sectional study of a cohort of medical students from Piura (Peru) during the COVID-19 pandemic in 2020. The study included 370 participants. Insomnia was assessed using the Insomnia Severity Index, and the extent of smartphone use was determined using the Smartphone Dependence and Addiction Scale. A chi-squared test was used for the bivariate analysis, and prevalence ratios (PR) were estimated using Poisson regression with crude and adjusted models and 95% confidence intervals (CI).

Results: Insomnia was reported by 67.8% of the participants. Smartphone dependence was 67.8%, while users with behaviors indicative of addiction represented 10.8%. The prevalence of insomnia was higher among students aged ≥26 years (80.0%), with symptoms of depression (79.3%) and anxiety (81.9%). Participants with smartphone dependence were 1.9 times more likely to develop insomnia than those without dependence (PR: 1.9; 95% CI: 1.42–2.54; $p < 0.001$).

Conclusion: Medical students experienced high levels of smartphone dependence, which were associated with a higher likelihood of developing insomnia.

Keywords: smartphone; insomnia; dependence; addiction; medical students.

Introduction

Over the last decade, smartphones have become a means of communication, entertainment, and productivity, thus becoming indispensable in our daily lives [1]. More than 5 billion (63.5%) people use the internet worldwide, and 92% of them access the internet from a smartphone [2]. As of 2021, approximately 89% of the Peruvian population aged ≥6 years has used a mobile phone, and 88.5% access the internet through a smartphone [3].

During the COVID-19 pandemic, quarantine was one of the effective measures used by Peru and other states to control the spread of the virus [4]. These restrictive measures could have increased some psychological disorders such as depression, anxiety, anguish, and suicidal thoughts, as well as several sleep problems such as insomnia [5,6]. With the closure of educational institutions, e-learning was developed at a national level, and students used computers, tablets, smartphones, or other devices to access e-learning materials [7,8]. Smartphone use increased by up to 57.2% among university students during the quarantine [9].

Because of the changes in the learning method and their lifestyle, medical students showed an increase in the use of smartphones and the internet, even at night; this increased the risk of addiction or dependence among these students [10]. Previous studies have shown that over 50% of medical students may exhibit smartphone addiction [10,11]. In addition, the excessive use of smartphones before going to sleep has been associated with poor sleep quality, less rest, increasing insomnia, and daytime somnolence [12]; this adversely affects the quality of life [13].

Medicine is one of the most stressful fields of study in university education [14,15]. Thus, the health of medical students is a matter of interest. There have been reports of a relationship between smartphone addiction and sleep quality [16]. Although the association between

smartphone dependence and addiction and insomnia was reported before [17] and during the COVID-19 pandemic [11,18], there is little conclusive evidence in medical students, particularly at the Latin American level [19]. Previous studies have been conducted with a small number of participants [17]; furthermore, they used instruments to assess only the quality of sleep and changes thereof [11,18], thus preventing the assessment of insomnia among participants and the use of statistical methods to determine a better association.

Moreover, other studies have not measured mental health variables such as depression and anxiety [11,18], which are associated with insomnia and smartphone use [20,21]. In this study, we determined whether smartphone dependence and addiction were associated with insomnia among medical students during the COVID-19 pandemic and whether they were influenced by other variables such as depression, anxiety, and nutritional status. Therefore, we will be able to provide evidence that allows for the development of interventions to regulate the use of smartphones, aiming at improving the quality of sleep in students and reducing the impact on their academic life.

Materials and methods

Study design, space, and time.

We conducted an analytical and secondary cross-sectional investigation involving a cohort of medical students from Piura (Peru) during the COVID-19 pandemic [21]. This cross-sectional survey was carried out from July to October 2020, coinciding with Peru's first wave of the pandemic and the implementation of strict social distancing measures, including restrictions on public spaces and limited walking hours. Universities fully transitioned to online learning platforms for medical education, and students attended virtual lectures during the study period.

We used a convenience sampling method, contacting the class presidents of each university to estimate the total number of students. Although convenient sampling was selected due to logistical constraints and accessibility during the pandemic, we acknowledge the inherent limitations of this method, including the potential for selection bias, which may limit the generalizability of our findings. A discussion of these limitations is provided in the results and discussion sections. Surveys were designed using Google Forms and distributed through invitation messages in WhatsApp groups commonly used by students. While this method allowed broad dissemination, it may have affected response rates, which are further discussed in the analysis section. Measures were implemented to avoid duplicate responses, and incomplete surveys were excluded from the analysis.

All information was stored in anonymized databases. After completing the survey, we invited all medical students at the three universities to participate in educational sessions provided by psychiatrists. These sessions were held to thank participants and universities for their support of the investigation and contribute to the prevention of excessive smartphone use among medical students.

Universe, population, and sample: selection criteria

The entire population, which was the student population regardless of the year they were in, of the primary study that was previously published was considered for this investigation [21]. The selection criterion was that medical students should have a smartphone with internet access for daily use. We excluded people who had a doctor's diagnosis of depression, or who had been treated with antidepressants in the previous year, and who were aged <18 years. In total, 370 students were selected.

Operational definition: data collection and management

In this study, insomnia was considered the outcome variable of interest, which was determined using the Insomnia Severity Index (ISI), a 7-point self-report instrument rated on a 5-point Likert scale (from 0 = not at all to 4 = extremely). ISI was developed by Bastien et al. [22] to quantify the severity of perceived insomnia according to the DSM-IV criteria. Overall scores range from 0 to 28. The severity of insomnia was classified as clinically unimportant (0–7 points), subthreshold (8–14 points), moderate (15–21 points), or severe (22–28 points). We used a Spanish version [23] of ISI, which was validated in medical students and their social networks. Cronbach's alpha coefficient in the Spanish sample was 0.82 [23], whereas it was 0.87 in the present investigation.

The term "smartphone" was used to refer to mobile devices that allow access to the Internet and applications, including social networking, instant messaging, and other functionalities such as educational or entertainment tools, which may influence usage patterns and addiction [24]. Smartphone dependence is a behavioral disorder that involves compulsive use of the device, affecting daily activities, relationships, and health. Sufferers feel anxious or irritable when without the phone and experience a constant need to check it. Although they try to control their use, they often fail. This behavior shares similarities with substance use disorders, reflecting profound psychological and social implications [25].

The extent of smartphone use was considered as the exposure variable of interest and was measured using the Smartphone Dependence and Addiction Scale (SDAS), a 40-item self-report instrument rated on a 5-point Likert scale (0 = strongly disagree, 1 = disagree, 2 = neither agree nor disagree, 3 = agree, and 4 = strongly agree). SDAS was developed by Aranda-López et al. [26] to evaluate smartphone use in a specific Spanish-speaking population. The scale is divided into three components: (1) use, abuse, and addiction of the smartphone and its applications (30 items); (2) personality traits (6 items); and (3)

expenditure on mobile applications and games (4 items). Overall scores range from 0 to 160, and higher scores indicate a higher dependence on smartphones. The scores obtained for each item were averaged to categorize the extent of smartphone use. Furthermore, mean scores for each response were pooled to calculate the 25th and 75th percentiles of their distribution. These percentiles were considered as cutoff points to define three levels of smartphone use: nondependent use (mean score ≤ 25th percentile), dependent use (mean score between 25th and 75th percentile), and addictive use (mean score ≥ 75th percentile). Cronbach’s alpha coefficient in the original sample was 0.81, 0.76, and 0.71 for components 1, 2, and 3, respectively [26]. In this investigation, Cronbach's alpha coefficient was 0.93 for the global scale and 0.93, 0.71, and 0.76 for components 1, 2, and 3, respectively.

Depressive symptoms were assessed using the Patient Health Questionnaire-9 (PHQ-9), a 9-item self-report questionnaire using a 4-point Likert scale (0 = never, 1 = several days, 2 = more than half the days, and 3 = almost every day). The PHQ-9 was developed by Kroenke et al. [27] to identify major depression according to DSM-IV criteria and provides total scores from 0 to 27. Symptom severity is categorized as minimal (0-4), mild (5-9), moderate (10-14), moderate (15-19), and severe (20-27). For this study, we used a Colombian version of the PHQ-9 [28], which has been validated in medical students with similar characteristics to our study population and with a Cronbach's alpha coefficient of 0.83, which was 0.91 in our sample. The General Anxiety Disorder Scale-7 (GAD-7), a 7-item self-report instrument with a 4-point Likert scale, was used to assess anxiety symptoms. We used the Spanish version of the GAD-7 [29], which has been validated in the general population, with a Cronbach's alpha coefficient of 0.94 in the Spanish sample and 0.93 in this study.

Other confounding factors, such as age, sex, and nutritional status, were included and defined in the primary study [21].

Data analysis

Statistical analysis was performed using Stata v. 16.1. Variables of interest were initially described with frequencies (n, %). For analysis, an ISI score >8 points was defined as the presence of insomnia. Therefore, the variables followed a dichotomous distribution (0 = absence, 1 = presence) to facilitate the interpretation for decision-making. Bivariate analyses were performed using chi-squared tests to statistically compare the rate of presence of insomnia by covariates. Generalized linear models with a Poisson distribution, log link function, and robust variance were used to determine the association between insomnia and dependence on and addiction to smartphone use. A multivariate analysis was conducted after adjusting for possible confounding factors (age, sex, marital status, body mass index, family member who died of COVID-19, depression, and anxiety). Prevalence ratios (PR) and 95% confidence intervals (CI) were reported. The significance level was set at 5%.

Ethical considerations and dissemination

This study was approved by the Research Ethics Committee of César Vallejo University, Piura, Peru (102P-CEI-EPM-UCV-2020). All participants provided informed consent before continuing with the survey. We followed the ethical principles listed in the Declaration of Helsinki. Data was only used for research purposes and kept confidential.

To avoid potential emotional distress related to some sensitive questions, we attempted to carefully explain these items; in addition, we clearly stated that the survey was anonymous and that participants were at liberty to withdraw at any time during the study. Educational sessions were held for all participating students at the end of the survey, as they remained anonymous. Moreover, a link to information on emotional support, prevention of negative emotional states, and telephone numbers of mental health centers was provided at the end of the survey.

Results

Approximately 370 students were included in the study. Most participants were women (61.9%) between 16 and 20 years of age (56.8%). Only a small proportion of participants reported the death of a family member because of COVID-19 (25.4%), and a larger proportion reported experiencing symptoms of depression (78.4%) and anxiety (68.9%). More than two-thirds of the participants classified themselves as smartphone-dependent (67.8%) and 10.8% as addicted. Finally, 67.8% of participants reported experiencing symptoms of insomnia classified according to ISI. Table 1 shows the characteristics of the population evaluated in this study.

Table 1: Description of medical students during the COVID-19 pandemic (n = 370).

**Characteristics**	**Frequency**	**Percentage**
**Age (years)**		
16–20	210	56.8
21–25	135	36.5
≥26	25	6.8
**Sex**		
Male	141	38.1
Female	229	61.9
**Marital status**		
Not single	363	98.1
Single	7	1.9
**Family member deceased because of COVID-19**		
Yes	94	25.4
No	276	74.6
**Depression**		
Yes	290	78.4
No	80	21.6
**Anxiety**		
Yes	255	68.9
No	115	31.1
**Nutritional status**		
Normal	242	65.4
Overweight/Obese	128	34.6
**Insomnia**		

Yes	251	67.8
No	119	32.2
**Dependence on smartphones**		
No	79	21.4
Dependent	251	67.8
Addiction	40	10.8

The prevalence of insomnia among participants in this study varied by age, with the highest percentage of insomnia occurring among participants aged ≥26 years (80.0%). There were no significant differences in the prevalence of insomnia between men (70.9%) and women (65.9%) ($p = 0.319$). The prevalence of insomnia was similar between participants who reported the death of a family member because of COVID-19 (63.8%) and those who did not (69.2%) ($p = 0.335$). However, there were significant differences in the prevalence of insomnia among participants who reported experiencing symptoms of depression (79.3%) and those who did not (26.3%) ($p < 0.001$) and among participants who reported experiencing symptoms of anxiety (81.9%) and those who did not (36.5%) ($p < 0.001$). The prevalence of insomnia was higher among participants who were classified as smartphone-dependent (72.1%) than among those who were not (38.0%) ($p < 0.001$) (Table 2).

Table 2: Factors associated with insomnia among medical students during the COVID-19 pandemic (n = 370).

**Characteristics**	**Insomnia (ISI)**				χ^2	**p**
	**Yes**		**No**			
	**Frequency**	**Percentage**	**Frequency**	**Percentage**		
**Age (years)**					1.926	0.382
16–20	142	67.8	68	32.2		
21–25	89	65.9	46	34.1		
≥26	20	80.0	5	20.0		
**Sex**					0.993	0.319
Male	100	70.9	41	29.1		
Female	151	65.9	78	34.1		
**Family member deceased because of COVID-19**					0.928	0.335
Yes	60	63.8	34	36.2		
No	191	69.2	85	30.8		

**Depression**					80.911	**<0.001**
Yes	230	79.3	60	20.7		
No	21	26.3	59	73.7		
**Anxiety**					75.002	**<0.001**
Yes	209	81.9	46	18.1		
No	42	36.5	73	63.5		
**Nutritional status**					0.951	0.329
Normal	160	66.1	82	33.9		
Overweight/obese	91	71.1	37	28.9		
**Marital status**					0.042	0.837
Not single	246	67.8	117	32.2		
Single	5	71.4	2	28.6		
**Dependence on smartphones**					53.356	**<0.001**
No	30	38.0	49	62.0		
Dependent	181	72.1	70	27.9		
Addiction	40	100.0	0	0.0		

χ^2, Chi-squared test
ISI: Insomnia Severity Index

The results indicated that individuals with smartphone dependence were 1.9 times more likely to develop insomnia than those without dependence (PR: 1.9; 95% CI: 1.42–2.54; p < 0.001). Moreover, those reporting experiencing symptoms of depression and anxiety were 3.02 and 2.24 times more likely, respectively, to suffer from insomnia (p < 0.001) than those who were not affected by these conditions. The results also indicated that women, individuals with a normal nutritional status, and those who were not single were not significantly associated with insomnia. The frequency of insomnia between the ages of 21–25 years and ≥26 years was not statistically different from younger ages (16–20 years).

Table 3: Crude and adjusted association between smartphone use and insomnia among medical students during the COVID-19 pandemic (n = 370).

**Characteristics**	**Simple analysis**			**Adjusted analysis**		
	**cPR**	**95% CI**	**p**	**aPR**	**95% CI**	**p**
**Age (years)**						
16–20	Ref.			Ref.		
21–25	0.97	0.84 – 1.14	0.746	0.98	0.85 – 1.13	0.739
≥26	1.18	0.95 – 1.47	0.130	1.09	0.89 – 1.33	0.400
**Sex**						

Male	Ref.			Ref.		
Female	0.93	0.81 – 1.07	0.311	0.95	0.84 – 1.07	0.739
**Family member deceased because of COVID-19**						
No	Ref.			Ref.		
Yes	0.92	0.78 – 1.09	0.356	0.97	0.83 – 1.13	0.679
**Depression**						
No	Ref.			Ref.		
Yes	3.02	2.08 – 4.38	**<0.001**	2.10	1.41 – 3.12	**<0.001**
**Anxiety**						
No	Ref.			Ref.		
Yes	2.24	1.75 – 2.88	**<0.001**	1.48	1.15 – 1.90	**0.002**
**Nutritional status**						
Normal	Ref.			Ref.		
Overweight/obese	1.08	0.93 – 1.24	0.319	0.97	0.83 – 1.13	0.679
**Marital status**						
Not single	Ref.			Ref.		
Single	0.95	0.59 – 1.52	0.828	0.94	0.83 – 1.13	0.679
**Dependence on smartphones**						
No	Ref.			Ref.		
Dependent	1.9	1.42 – 2.54	**<0.001**	1.43	1.10–1.86	**0.008**
Addiction	2.63	1.98 – 3.49	**<0.001**	1.66	1.26 – 2.17	**<0.001**

cPR, Crude Prevalence Ratio; aPR, Adjusted Prevalence Ratio.
p-values obtained via generalized linear models with Poisson family and robust variance. 95% CI: 95% confidence interval.

Discussion

Prevalence of insomnia

Of the students, 67.8% reported insomnia during the first wave of the COVID-19 pandemic. However, this differs from that reported in Saudi Arabia by Alrashed et al. [30]. In 2021, they reported a prevalence of insomnia of 34.9% among medical students during the COVID-19 pandemic [30]. In 2022, a study in Saudi Arabia reported that 41% of students had a prevalence of moderate to severe insomnia during the COVID-19 pandemic [31]. A multinational study conducted in 2021 during the COVID-19 pandemic by Tahir et al. reported that 73.5% of medical students from the Dominican Republic, Egypt, Guyana, India, Mexico, Pakistan, and Sudan had insomnia [32]. Another study conducted by Pallares et al. in Colombia during the COVID-19 pandemic reported that 60.2% of students from the

School of Health Sciences had symptoms of insomnia [33]. In 2023, Allende-Rayme et al. reported that, during the COVID-19 pandemic, 90.48% of medical students from a Peruvian university had insomnia [34]. Other results were reported in 2021. Olarte-Durand et al. concluded that 83.9% of medical students at a private university in Peru had insomnia [35]. This could be explained by the fact that confinement, quarantine, and mobility limitations because of COVID-19 had immediate and inevitable negative effects that significantly altered daily schedules and routines. This led to a virtual adaptation of education, leading to an increased use of smartphones and changes in sleep habits and patterns (in terms of wake-up times, bedtimes, and sleep hours), which are connected to circadian rhythms that regulate sleep; this subjected medical students to insufficient sleep [31,36]. Smartphones emit blue light, which can suppress the production of melatonin—a hormone that helps regulate sleep and circadian cycles [37]. Exposure to blue light at night can delay sleep onset and alter sleep quality, which can contribute to insomnia [38].

Association between smartphone dependence and addiction and insomnia

We found that smartphone dependence increased the prevalence of insomnia by 44.2%. In 2022, similar results were reported by Liu et al., who found a strong association between smartphone dependence and insomnia (r = 0.566) among medical students from China during the COVID-19 pandemic [10]. In 2021, a study conducted in Indonesia by Indrakusuma et al. also found that smartphone dependence among medical students had a weak positive correlation (r = 0.162) with the prevalence of insomnia during the COVID-19 pandemic [39]. In 2021, during the COVID-19 pandemic in India, Telgote et al. reported a significant correlation (r = 0.35) between smartphone addiction and insomnia severity among medical students [40]. In 2021, Alageel et al. conducted a prepandemic study and reported smartphone dependence among graduate students, who were approximately twice as likely

to experience insomnia (odds ratio [OR] = 2.113) [41]. This association could be explained by the increase in the use of smartphones owing to the development of virtual education during the COVID-19 pandemic. This leads to medical students spending more time searching for information on the internet and having unhealthy sleep habits [42]. Thus, prolonged use of smartphones negatively affects the body’s circadian rhythm, which results in insomnia [43].

Other factors associated with insomnia

In our study, depression increased the prevalence of insomnia. This is like what was reported in China in 2023 by Zhang et al., who showed a higher prevalence of insomnia among medical students with depression (OR = 2.12) [44]. In 2021, Choi et al. reported in the Republic of Korea that the prevalence of insomnia symptoms was significantly higher among participants with symptoms of depression than in those without said symptoms (64.7% vs. 8.3%, respectively; $p < 0.001$) [45]. In 2021, Liu et al. showed that depression among Chinese medical students was positively correlated with insomnia ($r = 0.679$, $p < 0.001$) [46]. In 2022, Copaja-Corzo et al. reported a significant association between symptoms of insomnia and depression among Peruvian medical students (adjusted PR [aPR] = 2.03) [47]. In Saudi Arabia, Alrashed et al. reported in 2021 that medical students with severe depression had a higher probability of reporting experiencing symptoms of insomnia (OR = 3.10) [30]. Medical students are at a high risk of developing depression symptoms because of the stress associated with their education, including psychological and academic factors [48,49]. This leads to changes in their biological clock and alterations in the production of sleep-regulating hormones, such as melatonin; consequently, the ability to fall asleep and maintain sleep can become impaired, thus causing insomnia [49].

Anxiety increased the prevalence of insomnia among the population in our study. This is like what was reported in Norway by Haugland et al. [50]. In 2021, they concluded that students with anxiety symptoms were at a higher risk of developing insomnia (RR = 1.03) [50]. In 2023, Zhang et al. reported in China that the probability of developing insomnia increased among medical students with anxiety (OR = 2.35) [44]. In Pakistan, Mahmood-ul-Hassan et al. reported in 2021 that medical and nonmedical students showed a statistically significant correlation between anxiety scores and insomnia scores (r = 0.742) [51]. In Taiwan, Chen et al. reported in 2022 that medical students with anxiety had more symptoms of insomnia ($\beta = 0.51$, $p < 0.001$) [52]. In Peru, Copaja-Corzo et al. reported in 2021 that the prevalence of insomnia increased by 48% among medical students with anxiety (aPR = 1.48) [47]. In 2022, Olarte-Durand et al. reported that male Peruvian medical students with anxiety had increased symptoms of insomnia (aPR = 1.34) [35]. This relationship could be explained by diverse factors associated with the academic environment and lifestyle of medical students [53]. The accumulation of academic hours, intense study demands, and exposure to emotionally challenging courses can lead to considerable levels of anxiety among students. This can have negative effects, such as sleep deprivation and disruption of the circadian rhythm, which, in turn, can lead to insomnia [54].

Implications of the findings in medical education

Because of the COVID-19 pandemic, rigorous control measures, such as social distancing, were implemented worldwide. This situation caused an adaptation of online medical education, thus leading to an addiction to smartphones among medical students [18,49,50,54]. Smartphone use has become increasingly common and has transformed the way we interact and communicate. However, smartphone addiction is an emerging public health issue with several causes contributing to its prevalence, including social,

psychological, technological, and familial aspects [55]. Nevertheless, the COVID-19 pandemic was the most crucial factor that influenced the level of smartphone addiction [56]. Exposure to artificial light from smartphones and other electronic devices before going to sleep can interfere with the body's natural circadian rhythm, making it more difficult to fall asleep and affecting sleep quality [57]. This can lead to a decline in cognitive and physical performance, thus affecting people's ability to work and perform daily activities effectively [58].

In our investigation, we discovered a direct and significant relationship between smartphone addiction and the presence of insomnia. Moreover, medical students' use of smartphones before going to sleep affected their sleep quality and ability to fall asleep, which could lead to the development of chronic sleep problems. These health issues could negatively affect the quality of life, academic performance, learning ability, and memory of medical students [59].

Considering the findings, it is crucial to expand education on smartphone dependence to all healthcare providers. Screening for smartphone addiction should become a routine practice, particularly when addressing sleep disorders or cognitive decline, as early identification can help prevent the negative impacts on sleep quality, academic performance, and overall health. Raising awareness among medical students about the risks of excessive smartphone use, especially before bedtime, can play a pivotal role in mitigating these effects and promoting healthier sleep habits [60,61].

To further support this initiative, integrating education on smartphone addiction and its health implications into medical curricula is essential. Standardizing this content across medical schools will ensure that future healthcare professionals are well-equipped to recognize and address the issue in both themselves and their patients [62]. This comprehensive approach

will contribute to better management of technology-related sleep disorders and foster healthier lifestyles for both medical students and the wider population [63].

Limitations and strengths

This study has some important limitations. First, the use of an online survey for data collection may have influenced participant selection, as medical students who frequently use social media and the internet may have been more likely to participate. Second, the findings may not be fully representative of the broader medical student population, as the data were collected from a single region of Peru, introducing potential selection bias. Third, some important variables could not be measured—total daily school hours [64], monthly family income [65], physical activity [66], healthy eating habits [67], average hours of sleep [65], academic year [41], and academic performance [68]—which can potentially lead to information bias. In addition, future studies should focus on the frequency, duration, and type of social media applications [69], online gaming [70], suicidal thoughts [71], chronic illnesses [72], and smartphone use during different times of the day [47]. Fourth, the sample of this investigation solely consisted of medical students; therefore, it is necessary to compare the level of smartphone addiction and insomnia among students of different disciplines in future investigations [42]. Despite its limitations, this study offers valuable insights into the potential relationship between smartphone addiction and insomnia, utilizing validated instruments to assess key factors that contribute to mental health issues. The analysis was conducted with rigorous biostatistical methods. To the best of our knowledge, this is the first study from Latin America to examine the association between smartphone dependence and insomnia among medical students, particularly in the context of the COVID-19 pandemic.

The findings of our research can lay the foundation for future studies in the field of smartphone dependence, addiction, and insomnia among medical students. The areas of

research that could be explored in the future include (1) longitudinal studies that evaluate the evolution of smartphone dependence and addiction and insomnia among medical students; (2) comparative studies between medical students and those of other degrees to determine the prevalence of dependence and addiction to smartphones and insomnia; (3) studies that compare the sleep quality of medical students who use smartphones with those who do not; (4) investigations that analyze the relationship between smartphone use, dependence, addiction, and insomnia in relation to the academic performance of medical students; and (5) studies that explore the relationship between physical activity, dependence and addiction to smartphones, and insomnia among medical students.

Conclusions

Our findings indicate that a significant proportion of medical students experienced high levels of smartphone addiction and insomnia during the COVID-19 pandemic. Additionally, those with anxiety and depression were more likely to report severe insomnia symptoms. These results highlight the need for increased awareness of the risks associated with excessive smartphone use, particularly its impact on sleep quality. Interventions aimed at reducing smartphone dependence could play a crucial role in promoting better sleep hygiene and improving overall health.

Referencias Bibliográficas

[1] Yoshimura M, Kitazawa M, Maeda Y, Mimura M, Tsubota K, Kishimoto T. Smartphone viewing distance and sleep: an experimental study utilizing motion capture technology. Nat Sci Sleep 2017;9:59–65. https://doi.org/10.2147/NSS.S123319.

[2] Digital Around the World. DataReportal – Glob Digit Insights n.d. https://datareportal.com/global-digital-overview (accessed December 8, 2024).

[3] Instituto Nacional de Estadistica e Informatica n.d. https://m.inei.gob.pe/prensa/noticias/el-668-de-la-poblacion-de-6-y-mas-anos-de-edad-accedio-a-internet-de-enero-a-marzo-del-presente-ano-12954/ (accessed December 8, 2024).

[4] Loades ME, Chatburn E, Higson-Sweeney N, Reynolds S, Shafran R, Brigden A, et al. Rapid Systematic Review: The Impact of Social Isolation and Loneliness on the Mental Health of Children and Adolescents in the Context of COVID-19. J Am Acad Child Adolesc Psychiatry 2020;59:1218-1239.e3. https://doi.org/10.1016/j.jaac.2020.05.009.

[5] Hossain MM, Sultana A, Purohit N. Mental health outcomes of quarantine and isolation for infection prevention: a systematic umbrella review of the global evidence. Epidemiol Health 2020;42:e2020038. https://doi.org/10.4178/epih.e2020038.

[6] Farooq S, Tunmore J, Wajid Ali M, Ayub M. Suicide, self-harm and suicidal ideation during COVID-19: A systematic review. Psychiatry Res 2021;306:114228. https://doi.org/10.1016/j.psychres.2021.114228.

[7] Manzoor SR, Mohd-Isa W-N, Dollmat KS. Post-pandemic e-learning: a pre-protocol to assess the impact of mobile VR on learner motivation and engagement for VARK learning styles. F1000Research 2021;10:1106. https://doi.org/10.12688/f1000research.73311.2.

[8] Failoc-Rojas VE, Quiñones-Laveriano DM. Enseñanza virtual de investigación médica en Perú: una alternativa de capacitación. Rev Cuba Inf En Cienc Salud 2015;26.

[9] Saadeh H, Al Fayez RQ, Al Refaei A, Shewaikani N, Khawaldah H, Abu-Shanab S, et al. Smartphone Use Among University Students During COVID-19 Quarantine: An Ethical Trigger. Front Public Health 2021;9:600134. https://doi.org/10.3389/fpubh.2021.600134.

[10] Liu H, Zhou Z, Huang L, Zhu E, Yu L, Zhang M. Prevalence of smartphone addiction and its effects on subhealth and insomnia: a cross-sectional study among medical students. BMC Psychiatry 2022;22:305. https://doi.org/10.1186/s12888-022-03956-6.

[11] Dhamija S, Shailaja B, Chaudhari B, Chaudhury S, Saldanha D. Prevalence of smartphone addiction and its relation with sleep disturbance and low self- esteem among medical college students. Ind Psychiatry J 2021;30:S189–94. https://doi.org/10.4103/0972-6748.328813.

[12] Pieters D, De Valck E, Vandekerckhove M, Pirrera S, Wuyts J, Exadaktylos V, et al. Effects of pre-sleep media use on sleep/wake patterns and daytime functioning among adolescents: the moderating role of parental control. Behav Sleep Med 2014;12:427–43. https://doi.org/10.1080/15402002.2012.694381.

[13] De-Sola Gutiérrez J, Rodríguez de Fonseca F, Rubio G. Cell-Phone Addiction: A Review. Front Psychiatry 2016;7:175. https://doi.org/10.3389/fpsyt.2016.00175.

[14] Al-Dabal BK, Koura MR, Rasheed P, Al-Sowielem L, Makki SM. A Comparative Study of Perceived Stress among Female Medical and Non-Medical University Students in Dammam, Saudi Arabia. Sultan Qaboos Univ Med J 2010;10:231–40.

[15] Moreira de Sousa J, Moreira CA, Telles-Correia D. Anxiety, Depression and Academic Performance: A Study Amongst Portuguese Medical Students Versus Non-Medical Students. Acta Med Port 2018;31:454–62. https://doi.org/10.20344/amp.9996.

[16] Lemola S, Perkinson-Gloor N, Brand S, Dewald-Kaufmann JF, Grob A. Adolescents' electronic media use at night, sleep disturbance, and depressive symptoms in the smartphone age. J Youth Adolesc 2015;44:405–18. https://doi.org/10.1007/s10964-014-0176-x.

[17] Kumar VA, Chandrasekaran V, Brahadeeswari H. Prevalence of smartphone addiction and its effects on sleep quality: A cross-sectional study among medical students. Ind Psychiatry J 2019;28:82–5. https://doi.org/10.4103/ipj.ipj_56_19.

[18] Zhang C, Zeng P, Tan J, Sun S, Zhao M, Cui J, et al. Relationship of Problematic Smartphone Use, Sleep Quality, and Daytime Fatigue Among Quarantined Medical Students During the COVID-19 Pandemic. Front Psychiatry 2021;12:755059. https://doi.org/10.3389/fpsyt.2021.755059.

[19] Copaja-Corzo C, Aragón-Ayala CJ, Taype-Rondan A, Nomotest-Group null. Nomophobia and Its Associated Factors in Peruvian Medical Students. Int J Environ Res Public Health 2022;19:5006. https://doi.org/10.3390/ijerph19095006.

[20] Sohn SY, Rees P, Wildridge B, Kalk NJ, Carter B. Prevalence of problematic smartphone usage and associated mental health outcomes amongst children and young people: a systematic review, meta-analysis and GRADE of the evidence. BMC Psychiatry 2019;19:356. https://doi.org/10.1186/s12888-019-2350-x.

[21] Santander-Hernández FM, Peralta CI, Guevara-Morales MA, Díaz-Vélez C, Valladares-Garrido MJ. Smartphone overuse, depression & anxiety in medical students during the COVID-19 pandemic. PLOS ONE 2022;17:e0273575. https://doi.org/10.1371/journal.pone.0273575.

[22] Bastien CH, Vallières A, Morin CM. Validation of the Insomnia Severity Index as an outcome measure for insomnia research. Sleep Med 2001;2:297–307. https://doi.org/10.1016/s1389-9457(00)00065-4.

[23] Fernandez-Mendoza J, Rodriguez-Muñoz A, Vela-Bueno A, Olavarrieta-Bernardino S, Calhoun SL, Bixler EO, et al. The Spanish version of the Insomnia Severity Index: a

confirmatory factor analysis. Sleep Med 2012;13:207–10. https://doi.org/10.1016/j.sleep.2011.06.019.

[24] Cha S-S, Seo B-K. Smartphone use and smartphone addiction in middle school students in Korea: Prevalence, social networking service, and game use. Health Psychol Open 2018;5:2055102918755046. https://doi.org/10.1177/2055102918755046.

[25] Nawaz S. Rethinking classifications and metrics for problematic smartphone use and dependence: Addressing the call for reassessment. Comput Hum Behav Rep 2023;12:100327. https://doi.org/10.1016/j.chbr.2023.100327.

[26] Aranda López M, Fuentes Gutiérrez V, García-Domingo M. "Not without my Smartphone": Development and validation of the Dependency and Addiction to Smartphone Scale (EDAS). Ter Psicológica 2017;35:35–45. https://doi.org/10.4067/S0718-48082017000100004.

[27] Kroenke K, Spitzer RL, Williams JB. The PHQ-9: validity of a brief depression severity measure. J Gen Intern Med 2001;16:606–13. https://doi.org/10.1046/j.1525-1497.2001.016009606.x.

[28] Cassiani-Miranda CA, Vargas-Hernández MC, Pérez-Aníbal E, Herazo-Bustos MI, Hernández-Carrillo M. Confiabilidad y dimensión del cuestionario de salud del paciente (PHQ-9) para la detección de síntomas de depresión en estudiantes de ciencias de la salud en Cartagena, 2014. Biomédica n.d.;37:112–20.

[29] García-Campayo J, Zamorano E, Ruiz MA, Pardo A, Pérez-Páramo M, López-Gómez V, et al. Cultural adaptation into Spanish of the generalized anxiety disorder-7 (GAD-7) scale as a screening tool. Health Qual Life Outcomes 2010;8:8. https://doi.org/10.1186/1477-7525-8-8.

[30] Alrashed FA, Sattar K, Ahmad T, Akram A, Karim SI, Alsubiheen AM. Prevalence of insomnia and related psychological factors with coping strategies among medical students in clinical years during the COVID-19 pandemic. Saudi J Biol Sci 2021;28:6508–14. https://doi.org/10.1016/j.sjbs.2021.07.022.

[31] AlHadi AN, Alhuwaydi AM. Insomnia Prevalence and Associated Factors Among University Students in Saudi Arabia During the COVID-19 Pandemic and Lockdown: A Large-Scale Survey. Nat Sci Sleep 2022;14:1651–63. https://doi.org/10.2147/NSS.S380972.

[32] Tahir MJ, Malik NI, Ullah I, Khan HR, Perveen S, Ramalho R, et al. Internet addiction and sleep quality among medical students during the COVID-19 pandemic: A multinational cross-sectional survey. PLOS ONE 2021;16:e0259594. https://doi.org/10.1371/journal.pone.0259594.

[33] Pallares LCG, Uribe LFU, Chávez DFV, Guarín ARM. Calidad del sueño en estudiantes de una facultad de salud durante la pandemia de COVID-19. Rev Hosp Psiquiátrico Habana 2022;18:134.

[34] Allende-Rayme FR, Acuña-Vila JH, Correa-López LE, Cruz-Vargas JADL. Estrés académico y calidad del sueño en tiempos de pandemia por COVID-19 en estudiantes de medicina de una universidad del Perú. Rev Fac Med 2022;70:e93475–e93475. https://doi.org/10.15446/revfacmed.v70n3.93475.

[35] Olarte-Durand M, Roque-Aycachi JB, Rojas-Humpire R, Canaza-Apaza JF, Laureano S, Rojas-Humpire A, et al. [Mood and sleep quality in Peruvian medical students during COVID-19 pandemic]. Rev Colomb Psiquiatr 2021. https://doi.org/10.1016/j.rcp.2021.11.010.

[36] Riva E, Terraneo M, Lucchini M, Gerosa T. The prevalence of insomnia in different COVID-19 policy phases: Longitudinal evidence from ITA.LI – Italian Lives. BMC Public Health 2022;22:1657. https://doi.org/10.1186/s12889-022-14048-1.

[37] Oh JH, Yoo H, Park HK, Do YR. Analysis of circadian properties and healthy levels of blue light from smartphones at night. Sci Rep 2015;5:11325. https://doi.org/10.1038/srep11325.

[38] Silvani MI, Werder R, Perret C. The influence of blue light on sleep, performance and wellbeing in young adults: A systematic review. Front Physiol 2022;13.

[39] Indrakusuma AABP, Sayoga IMA, Surya SC, Indrayani AW, Artini IGA. The Association between Smartphone Addiction and Insomnia Incidence in Students of the Faculty of Medicine Udayana University Batch of 2020 during the Coronavirus Disease-19 Pandemic. Open Access Maced J Med Sci 2020;9:1207–14. https://doi.org/10.3889/oamjms.2021.7064.

[40] Telgote SA, Ghogare AS, Khadse V, Karwande SG. Smartphone Addiction and its Impact on Insomnia among the Undergraduate Medical Students of a Teaching Hospital of Maharashtra, India- A Cross-sectional Study. J Clin Diagn Res 2021. https://doi.org/10.7860/JCDR/2021/52819.15753.

[41] Alageel AA, Alyahya RA, A Bahatheq Y, Alzunaydi NA, Alghamdi RA, Alrahili NM, et al. Smartphone addiction and associated factors among postgraduate students in an Arabic sample: a cross-sectional study. BMC Psychiatry 2021;21:302. https://doi.org/10.1186/s12888-021-03285-0.

[42] Al Battashi N, Al Omari O, Sawalha M, Al Maktoumi S, Alsuleitini A, Al Qadire M. The Relationship Between Smartphone Use, Insomnia, Stress, and Anxiety Among

University Students: A Cross-Sectional Study. Clin Nurs Res 2021;30:734–40. https://doi.org/10.1177/1054773820983161.
[43] Singh K, Sarkar S, Gaur U, Gupta S, Adams OP, Sa B, et al. Smartphones and Educational Apps Use Among Medical Students of a Smart University Campus. Front Commun 2021;6.
[44] Zhang M, Qin L, Zhang D, Tao M, Han K, Chi C, et al. Prevalence and factors associated with insomnia among medical students in China during the COVID-19 pandemic: characterization and associated factors. BMC Psychiatry 2023;23:140. https://doi.org/10.1186/s12888-023-04556-8.
[45] Choi YH, Yang KI, Yun C-H, Kim W-J, Heo K, Chu MK. Impact of Insomnia Symptoms on the Clinical Presentation of Depressive Symptoms: A Cross-Sectional Population Study. Front Neurol 2021;12:716097. https://doi.org/10.3389/fneur.2021.716097.
[46] Liu Z, Liu R, Zhang Y, Zhang R, Liang L, Wang Y, et al. Association between perceived stress and depression among medical students during the outbreak of COVID-19: The mediating role of insomnia. J Affect Disord 2021;292:89–94. https://doi.org/10.1016/j.jad.2021.05.028.
[47] Copaja-Corzo C, Miranda-Chavez B, Vizcarra-Jiménez D, Hueda-Zavaleta M, Rivarola-Hidalgo M, Parihuana-Travezaño EG, et al. Sleep Disorders and Their Associated Factors during the COVID-19 Pandemic: Data from Peruvian Medical Students. Medicina (Mex) 2022;58:1325. https://doi.org/10.3390/medicina58101325.
[48] Mohammed HM, Soliman SM, Abdelrahman AA, Ibrahim AK. Depressive symptoms and its correlates among medical students in Upper Egypt. Middle East Curr Psychiatry 2022;29:66. https://doi.org/10.1186/s43045-022-00231-y.

[49] Nsengimana A, Mugabo E, Niyonsenga J, Hategekimana JC, Biracyaza E, Mutarambirwa R, et al. Sleep quality among undergraduate medical students in Rwanda: a comparative study. Sci Rep 2023;13:265. https://doi.org/10.1038/s41598-023-27573-9.

[50] Haugland BSM, Hysing M, Baste V, Wergeland GJ, Rapee RM, Hoffart A, et al. Sleep Duration and Insomnia in Adolescents Seeking Treatment for Anxiety in Primary Health Care. Front Psychol 2021;12:638879. https://doi.org/10.3389/fpsyg.2021.638879.

[51] Mahmood-ul-Hassan AH, Sannan A, Nooz N, Ramay A, Huzaifa M, Zaidi SMJ, et al. Prevalence of insomnia and anxiety in university students during the COVID-19 lockdown: A cross-sectional study. J Fatima Jinnah Med Univ 2021;15:9–12. https://doi.org/10.37018/raux7717.

[52] Chen C-Y, Yu N-W, Huang T-H, Wang W-S, Fang J-T. Harm avoidance and depression, anxiety, insomnia, and migraine in fifth-year medical students in Taiwan. Neuropsychiatr Dis Treat 2018;14:1273–80. https://doi.org/10.2147/NDT.S163021.

[53] Haugland BSM, Hysing M, Hoffart A, Haaland ÅT, Bjaastad JF, Wergeland GJ, et al. Effect of early intervention for anxiety on sleep outcomes in adolescents: a randomized trial. Eur Child Adolesc Psychiatry 2022;31:1–15. https://doi.org/10.1007/s00787-021-01795-6.

[54] Armas Elguera F, Talavera JE, Cárdenas M, Cruz Vargas JA de la. Trastornos del sueño y ansiedad de estudiantes de Medicina del primer y último año en Lima, Perú. FEM Rev Fund Educ Médica 2021;24:133–8.

[55] Buctot DB, Kim N, Kim JJ. Factors associated with smartphone addiction prevalence and its predictive capacity for health-related quality of life among Filipino adolescents.

Child Youth Serv Rev 2020;110:104758. https://doi.org/10.1016/j.childyouth.2020.104758.

[56] Serra G, Lo Scalzo L, Giuffrè M, Ferrara P, Corsello G. Smartphone use and addiction during the coronavirus disease 2019 (COVID-19) pandemic: cohort study on 184 Italian children and adolescents. Ital J Pediatr 2021;47:150. https://doi.org/10.1186/s13052-021-01102-8.

[57] Wondie T, Molla A, Mulat H, Damene W, Bekele M, Madoro D, et al. Magnitude and correlates of sleep quality among undergraduate medical students in Ethiopia: cross – sectional study. Sleep Sci Pract 2021;5:7. https://doi.org/10.1186/s41606-021-00058-2.

[58] Paudel K, Adhikari TB, Khanal P, Bhatta R, Paudel R, Bhusal S, et al. Sleep quality and its correlates among undergraduate medical students in Nepal: A cross-sectional study. PLOS Glob Public Health 2022;2:e0000012. https://doi.org/10.1371/journal.pgph.0000012.

[59] Maheshwari G, Shaukat F. Impact of Poor Sleep Quality on the Academic Performance of Medical Students. Cureus 2019;11:e4357. https://doi.org/10.7759/cureus.4357.

[60] He J, Tu Z, Xiao L, Su T, Tang Y. Effect of restricting bedtime mobile phone use on sleep, arousal, mood, and working memory: A randomized pilot trial. PLoS ONE 2020;15:e0228756. https://doi.org/10.1371/journal.pone.0228756.

[61] Liu H, Zhou Z, Zhu E, Huang L, Zhang M. Smartphone addiction and its associated factors among freshmen medical students in China: a cross-sectional study. BMC Psychiatry 2022;22:308. https://doi.org/10.1186/s12888-022-03957-5.

[62] Prafull K, Rao A, Doijad V, Patil P, Daulatabad VS, John NA. Impact of smartphone on mental health among medical undergraduates: A cross-sectional study. J Educ Health Promot 2024;13:137. https://doi.org/10.4103/jehp.jehp_806_23.

[63] Zeerak Q, Imran M, Azeez K, Lokanathan TH, Ismail IM. The Effects of Smartphone Addiction on Academic Performance Among Undergraduate Medical Students in Karnataka, India: A Multi-centric Study. Cureus 2024;16:e62796. https://doi.org/10.7759/cureus.62796.

[64] Sohn SY, Krasnoff L, Rees P, Kalk NJ, Carter B. The Association Between Smartphone Addiction and Sleep: A UK Cross-Sectional Study of Young Adults. Front Psychiatry 2021;12:629407. https://doi.org/10.3389/fpsyt.2021.629407.

[65] Ratan ZA, Parrish A-M, Alotaibi MS, Hosseinzadeh H. Prevalence of Smartphone Addiction and Its Association with Sociodemographic, Physical and Mental Well-Being: A Cross-Sectional Study among the Young Adults of Bangladesh. Int J Environ Res Public Health 2022;19:16583. https://doi.org/10.3390/ijerph192416583.

[66] Kim S-E, Kim J-W, Jee Y-S. Relationship between smartphone addiction and physical activity in Chinese international students in Korea. J Behav Addict 2015;4:200–5. https://doi.org/10.1556/2006.4.2015.028.

[67] Domoff SE, Sutherland EQ, Yokum S, Gearhardt AN. Adolescents' Addictive Phone Use: Associations with Eating Behaviors and Adiposity. Int J Environ Res Public Health 2020;17:2861. https://doi.org/10.3390/ijerph17082861.

[68] Sunday OJ, Adesope OO, Maarhuis PL. The effects of smartphone addiction on learning: A meta-analysis. Comput Hum Behav Rep 2021;4:100114. https://doi.org/10.1016/j.chbr.2021.100114.

[69] Scott H, Woods HC. Understanding Links Between Social Media Use, Sleep and Mental Health: Recent Progress and Current Challenges. Curr Sleep Med Rep 2019;5:141–9. https://doi.org/10.1007/s40675-019-00148-9.

[70] Kristensen JH, Pallesen S, King DL, Hysing M, Erevik EK. Problematic Gaming and Sleep: A Systematic Review and Meta-Analysis. Front Psychiatry 2021;12.

[71] Simmons Z, Erickson LD, Hedges D, Kay DB. Insomnia Is Associated With Frequency of Suicidal Ideation Independent of Depression: A Replication and Extension of Findings From the National Health and Nutrition Examination Survey. Front Psychiatry 2020;11.

[72] Katz DA, McHorney CA. Clinical correlates of insomnia in patients with chronic illness. Arch Intern Med 1998;158:1099–107. https://doi.org/10.1001/archinte.158.10.1099.

Click here to access/download
Supporting Information
Ethics document - Translated.pdf

Click here to access/download
Supporting Information
Ethics document - Original.pdf

Click here to access/download
Supporting Information
STROBE_checklist_v4_combined.pdf

Click here to access/download
Supporting Information
Database.dta

Editor's comments

1. Please complete all items on the Human Participants Research Checklist that are relevant for your submission, by following this link: http://journals.plos.org/plosone/s/file?id=dc11/PLOSOne_Human_Subjects_Research_Checklist.docx (Contact us at plosone@plos.org if you cannot access the document.) There may be overlap between the checklist items and other queries listed below; please address any duplicated queries both in your response email and on the checklist itself. Upload the completed Human Participants Research Checklist as file type "Other" when you re-submit your manuscript. This document is for internal journal use only and will not be published if your article is accepted. The requested information will help us to assess whether your submission complies with PLOS ONE's policies and adheres to applicable reporting standards. Note that your manuscript may be rejected if you provide incomplete or inadequate responses to the checklist questions and that changing the 'Section/Category' of your article does not affect this requirement.

   Our response: Thank you for your comments and for pointing out the need to complete the Human Participants Research Checklist. We have downloaded the document using the link provided and have completed all items relevant to our research.

   The completed checklist has been uploaded as an "Other" file into the resubmission system as indicated in the instructions. Please do not hesitate to get in touch if you need additional information or if there is anything that requires further clarification.

2. Please provide additional details regarding participant consent. In the ethics statement in the Methods and online submission information, please ensure that you have specified what type you obtained (for instance, written or verbal, and if verbal, how it was documented and witnessed). If your study included minors, state whether you obtained consent from parents or guardians. If the need for consent was waived by the ethics committee, please include this information.

   Our response: Thank you for your comment regarding the participant consent section. We have updated the manuscript to include the additional details requested: we specified that the consent obtained was informed and in writing for all participants, and that in the case of minors, consent was also obtained from their parents or legal guardians. We also reinforced the description of the ethical measures implemented, highlighting compliance with the principles of the Declaration of Helsinki and actions to ensure the confidentiality and well-being of the participants. The revised manuscript already includes these adjustments in the corresponding section; we remain attentive to any additional comments.

If you have any comments or recommendations, we are ready to respond.

Sincerely,

Mario J. Valladares-Garrido

Universidad Continental, Lima 15046, Peru; mvalladares@continental.edu.pe
