# Supplementary material for: Smartphone dependence, addiction, and insomnia among medical students during the COVID-19 pandemic
Source: PLoS One. 2025 Jul 31;20(7):e0329495. doi: 10.1371/journal.pone.0329495 (PMC12312953; doi:10.1371/journal.pone.0329495)
Supplement: Attachment — Submitted filename: Response to Reviewers.pdf [file pone.0329495.s005.pdf]

Dear **Editor**,

Thank you very much for reviewing our article, " ***Smartphone dependence, addiction, and insomnia among medical students during the COVID-19 pandemic*** ". Your suggestions and comments will be addressed below. Thank you for your valuable time and excellent review.

Editor's comments

1. **Editor says:** We suggest expanding the introduction to include additional literature on behavioral addiction and its relationship with mental health conditions. This will help contextualize the study within the broader research landscape and better articulate the research gap your study aims to address. Highlighting the novelty of your approach and findings will strengthen the manuscript further.
   Our response: Thank you for your comment. As suggested, the introduction has been expanded with literature on smartphone addiction and its association with anxiety and depression.

2. **Editor says:** In the methods section, please provide greater detail on the sampling strategy, including the number of universities selected, and elaborate on the exclusion process that led to the final sample size. It would also be helpful to clarify how confounding variables, such as nutritional status, were identified and measured, as well as how the adjustments were applied during the analysis. Additionally, we recommend reorganizing the section to separate the description of scales and instruments from operational definitions, which could be included as a distinct subsection for improved readability.

   Our response: Thank you for your insightful comments. We appreciate the opportunity to clarify and enhance the methodology section. We have addressed each point as follows:

a) **Ethical considerations (form of consent)**: We have added more detailed information regarding the form of consent obtained, as follows: "All participants provided virtual informed consent before proceeding with the survey. This was achieved through a consent form embedded at the beginning of the online questionnaire. Participants were required to read and agree to the consent terms before accessing the survey questions." (lines 398-416).

b) **Sampling strategy and universities selected**: Three universities were selected for this study, all offering the human medicine program in the Piura region, one of the most affected areas in terms of COVID-19 morbidity and mortality in Peru. The universities were chosen based on availability and willingness to participate, with no specific sampling strategy. The sampling approach was non-probabilistic and based on convenience, given the logistical constraints posed by the pandemic. This is now clarified in lines 201-207.

c) **Exclusion criteria (number of participants excluded)**: The survey initially included a larger number of participants, but 38 were excluded due to a history of depression or anxiety, diagnosed by a healthcare professional or treated with antidepressants/anxiolytics in the previous year. This resulted in a final sample size of 370 participants. This is now included in lines 198-207.

d) **Confounding variables (identification and adjustment)**: Confounding variables were selected based on prior literature, expert opinion, and known relationships with the outcome variables (insomnia and smartphone dependence/addiction). A stepwise approach was used to determine the minimal set of confounders, including age, sex, marital status, BMI, family member deceased due to COVID-19, depression, and anxiety. These variables were tested for inclusion based on statistical significance. This is now clarified in lines 323-382.

e) **Separation of scales/instruments and operational definitions**: As requested, we have reorganized the methods section to clearly separate the description of scales from the operational definitions. The description of each instrument (Insomnia Severity Index, Smartphone Dependence and Addiction Scale, PHQ-9, and GAD-7) is now in a separate subsection, with operational definitions of the variables of interest placed at the end of the methods section, as suggested.

We hope these changes address your concerns and improve the clarity and quality of the manuscript.

3. **Editor says:** The ethical considerations require clarification, specifically regarding the type of consent obtained from participants. This information should be included explicitly to align with ethical guidelines and ensure transparency.
Our response: Thank you for your valuable comment. We appreciate your suggestion to clarify the ethical considerations, particularly regarding the type of consent obtained from participants. In response to this, we have expanded the Ethical Considerations section to provide a more comprehensive description of the consent process.

We now specify that virtual informed consent was obtained from all participants before they proceeded with the survey. The consent process was clearly outlined at the beginning of the online questionnaire, where participants were presented with a detailed consent form. This form included comprehensive information about the study's purpose, procedures, potential risks, confidentiality, and their right to withdraw at any time without consequence. Participants were required to read the consent form carefully and electronically agree to the terms by selecting an option that indicated their consent before gaining access to the survey questions. This method ensured that all participants had a clear understanding of the study and voluntarily agreed to participate.

4. **Editor says:** In the results section, address the discrepancies between the methods and results regarding the categorization of variables such as depression and anxiety. Additionally, refine the interpretation of prevalence ratios to provide a clearer understanding of the findings.
Our response: Thank you for your insightful comment. We apologize for the oversight in the methods section. To clarify, both depression and anxiety were treated as categorical variables based on symptom severity, as follows:

- **Depression**: Symptoms were categorized into minimal (0-4), mild (5-9), moderate (10-14), moderately severe (15-19), and severe (20-27). For analysis, we dichotomized it into two categories: no depression (0-4) and depressive symptoms (5+).
- **Anxiety**: Symptoms were categorized into minimal (0-4), mild (5-9), moderate (10-14), and severe (15-21). Similarly, we dichotomized it into no anxiety (0-4) and anxiety symptoms (5+).

We hope this clarification resolves the discrepancies and improves the interpretation of the results.

5. **Editor says:** Finally, the discussion would benefit from a deeper reflection on the implications of your findings in addressing the research gap and contributing to future work in this area. Explicit acknowledgment of the methodological limitations, including potential selection biases and the rationale for excluding certain clinical conditions, will enhance the manuscript's rigor and balance.

   Our response:

   Thank you for your insightful comments and for the opportunity to improve our manuscript. We appreciate your suggestions regarding the discussion section and have made the following revisions:

   - We have expanded our discussion on how our findings address the research gap. Specifically, we highlight how our study provides novel insights into smartphone addiction and insomnia among medical students in Latin America, a population that has been underrepresented in previous research.
   - We have further elaborated on how our findings can inform future research by suggesting potential studies that explore causal mechanisms, intervention strategies, and comparative analyses across different student populations.
   - We have explicitly acknowledged additional methodological limitations, including a more detailed discussion on potential selection bias due to the online survey format and the rationale for excluding certain clinical conditions.

Reviewer #1:

1. **Reviewer says:** Please read the attached annotated manuscript for comments. The manuscript details findings on associations between various count data variables, namely, smartphone dependence and insomnia and associated sociodemographic variables. Whilst there is nothing inherently problematic in the manuscript, the amount of new information to the area is adequate.
   Our response: Thank you for your valuable feedback. We acknowledge that the associations between smartphone dependence, insomnia, and sociodemographic variables, such as student stress, have been well-documented in the literature. However, we believe that our study provides significant value by focusing on medical students during the pandemic, a population that faced unique challenges related to the rapid transition to online learning, increased mental health burdens, and changes in daily routines. The analytical approach we used allowed us to examine these variables in a comprehensive way, providing insight into the specific impact of the pandemic on this group. Our study also contributes to the existing literature by considering the unique challenges faced by medical students in Piura, a region in northern Peru, especially regarding the transition to remote learning during the COVID-19 pandemic. Additionally, we employed a cross-sectional analytical approach, utilizing validated self-report scales to assess smartphone dependence and insomnia, which allows for a more nuanced understanding of these issues in a specific cohort. While the findings may not be novel, we hope to contribute to a better understanding of these issues and their implications for medical education and student well-being during extraordinary circumstances. Furthermore, we have carefully applied statistical analyses to ensure the data is interpreted in the most accurate and meaningful way possible.

Finally, we have emphasized these points in the **Implications of Findings** section of the manuscript to highlight the unique context of medical students during the pandemic. Moreover, we recognize the limitations of our study in the **Discussion** section, acknowledging factors such as the cross-sectional design and the self-reported nature of the data, which may limit the generalizability of the findings.

2. **Reviewer says:** Self-reported student stress and smartphone time consumption are significantly associated but this finding is not novel nor unexecpted. Nonetheless, the research design and statistical analyses is correct, and the authors do not move beyond what the data suggests.

   Our response:
   We sincerely appreciate the reviewer's insightful comments, which have helped us refine our manuscript. In response, we have strengthened the discussion by highlighting our study's contribution to existing knowledge and its broader implications. Specifically, we have expanded the discussion on how our findings add new empirical evidence from a Latin American population and complement previous studies conducted in other regions.
   Please find the revised manuscript attached. We hope the changes meet the reviewer's expectations.

Reviewer #2:

1. **Reviewer says:** In the introduction section, authors have mentioned limited literature related to the study, they are suggested to add more on behavioral addiction and smart phone addition and its association with mental health conditions.
   Our response: Thank you for your comment. As suggested, I have added literature on smartphone addiction and its association to anxiety and depressive symptoms.

   In method section (line no. 97-100), authors mentioned "we acknowledge the inherent limitations of this method, including the potential for selection bias, which may limit the generalizability of our findings. A discussion of these limitations is provided in the results and discussion sections." This should only be mentioned in the limitation section of the study.
   Our response: Thank you for your helpful comment. We apologize for the oversight in the methods section. As suggested, we have removed the mention of selection bias and its potential impact on the generalizability of our findings from the methods section. This information has now been properly included in the limitations section of the manuscript.

2. **Reviewer says:** Line 114 and 115, authors have mentioned that "they excluded people who had a doctor's diagnosis of depression, or who had been treated with antidepressants in the previous year. How about anxiety and other clinical conditions, why only excluding depression. As we know that during pandemic more anxiety was reported then depression, kindly clarify this.
   Our response: Thank you for pointing out this important detail. We apologize for the error in the initial version of the manuscript. In fact, both depression and anxiety were considered exclusion criteria for participation in the study due to their potential impact

on the participants' mental health status and the risk of bias in the study outcomes. This has now been clarified and reformulated in lines 115-119 of the methods section.

3. **Reviewer says:** Authors are requested to give description of scales/instruments in subsection separately. They are requested to mention the operational definitions separately at the end of the method section, it should not be mixed up with the description of scales/instruments.
   Our response: We appreciate the reviewer's observation. As requested, we have reorganized the information in the methods section to clearly separate the description of the scales from the operational definitions. The description of each instrument (Insomnia Severity Index, Smartphone Dependence and Addiction Scale, PHQ-9, and GAD-7) is now located in a separate subsection, while the operational definitions of the variables of interest (insomnia, depressive and anxiety symptoms) have been placed at the end of the methods section, as requested.

Reviewer #3:

1. **Reviewer says:** 1. In Ethics statement, please "indicate the form of consent obtained"
2. 2. Please find the comments for given line numbers below:
   Our response: Thank you for your valuable comment. We have added more detailed information regarding the form of consent obtained as follows***:*** "All participants provided virtual informed consent before proceeding with the survey. This was achieved through a consent form embedded at the beginning of the online questionnaire. Participants were required to read and agree to the consent terms before accessing the survey questions. ***(lines 398-416).***

3. **Reviewer says:** Line 95. How many universities were selected? Were all universities in Peru included? If not, what sampling strategy was used?
   Our response: Thank you for your comment. Three universities were selected for this study, all of which offer the human medicine program in the Piura region, one of the most affected areas in terms of COVID-19 morbidity and mortality in Peru. These universities were selected based on their availability and willingness to participate, and no specific sampling strategy was used to select them.
   The sampling approach used for this study was non-probabilistic and based on convenience, which was the most feasible option given the logistical constraints and limitations posed by the pandemic. We acknowledge the potential limitations of this approach, including the possibility of selection bias, which may affect the generalizability of the findings. These limitations are discussed further in the results and discussion sections. This has been included in **lines 173-207** of the Methods section.

4. **Reviewer says:** Line 116. How many participants were included in the survey? And how many were excluded to reach the final sample size of 370?
   Our response: The survey included a total of 370 participants, representing a subset of the medical student population across three universities. Of the initial participants, 38 were excluded due to a history of depression/anxiety, either diagnosed by a healthcare professional or having been treated with antidepressants or anxiolytics in the previous year. The final sample size of 370 participants was reached after applying this exclusion criterion. This information is discussed in detail between **lines 198-207** of the Methods section.

5. **Reviewer says:** Line 157. I suppose, 15-19 should be categorized as 'moderately severe' instead of 'moderate'.
Our response: Thank you for your suggestion. We have revised the categorization as per your recommendation. The range of 15-19 has now been categorized as "moderately severe" instead of "moderate." This change has been reflected in line 279 of the manuscript.

6. **Reviewer says**: Line 164. Details on confounding variables could be elaborated. For example, how was the nutritional status measured? Also, were the assumptions of poisson regression met?
Our response: We thank the reviewer for the suggestion to provide more details on the confounding variables (lines 390-397). Regarding the nutritional status of the participants, this was measured through self-reported weight and height, and based on these data, the Body Mass Index (BMI) was estimated. The participants were then classified into different categories according to the standard BMI ranges: underweight, normal weight, overweight, and obesity.

   Regarding the use of Poisson regression, to ensure model convergence and allow for the estimation of prevalence ratios (PR), robust variance was used. This approach helped manage potential correlations and heteroscedasticity in the data, ensuring more accurate estimates of the model parameters. This detail has been included in the statistical analysis section of the manuscript. (Lines 389-397).

7. **Reviewer says:** Line 175. How were the confounding variables identified, and how was the minimal set of adjusted confounders determine? (e.g. using DAG or any models)
Our response: We appreciate the reviewer's suggestion regarding the identification of confounding variables. The confounding variables were selected based on a combination of prior literature, expert opinion, and known relationships between the variables and the outcome of interest (insomnia and smartphone dependence/addiction). The potential confounders were identified through a review of existing research on mental health and smartphone use, as well as the clinical judgment of the research team.
The minimal set of adjusted confounders was determined through a stepwise approach, where we first identified variables that were known to have a potential association with both the outcome and predictor (smartphone dependence/addiction). These included age, sex, marital status, body mass index (BMI), family member deceased due to COVID-19, depression, and anxiety. These variables were then tested for inclusion in the final model based on their statistical significance and relevance to the study's hypothesis.

8. **Reviewer says**: Line 180. I suppose, the participants provided a written consent.
Our response: We apologize for the oversight and thank you for your valuable comment. We have corrected the statement to clarify that the participants provided virtual informed consent before proceeding with the survey **(lines 398-403)**. This was done through an embedded consent form at the beginning of the online questionnaire.

9. **Reviewer says:** Line 199. In the methods section, it says that depression was a categorical variable. However, in the results, it is presented as a dichotomous variable. Could you explain how it was dichotomized? Same for anxiety, the methods section does not explain how the variable anxiety was treated.

Our response: Thank you for your insightful comment. We apologize for the oversight in the methods section. To clarify, both depression and anxiety were treated as categorical variables based on symptom severity, and we provide further details below:

- For depression, the severity of symptoms was categorized as minimal (0-4), mild (5-9), moderate (10-14), moderately severe (15-19), and severe (20-27) using a standardized scale. For analysis, we dichotomized the variable into two categories: participants with scores of 0-4 were considered as having no depression, and those with scores of 5 or more were categorized as having depressive symptoms.
- For anxiety, the symptom severity was categorized as minimal (0-4), mild (5-9), moderate (10-14), and severe (15-21). Similarly, the anxiety variable was dichotomized for the analysis: participants with scores of 0-4 were considered as not having anxiety symptoms, while those with scores of 5 or more were categorized as having anxiety symptoms.

10. **Reviewer says:** Line 217. Since you are reporting exp beta, an interpretation like this "The prevalence of insomnia was 1.9 times higher among individuals with smartphone dependence compared to those without dependence" may be better than saying "more likely to" (as it sounds more like interpreting likelihood)

    Our response:
    Thank you for your comments, we have improved the interpretation of the exp beta with more appropriate terms in the results section.

11. **Reviewer says:** Line 223. Since you are talking about the frequency of insomnia, are you referring to table 2?

    Our response:
    We refer to table 3, this has been described in the text.

If you have any comments or recommendations, we are ready to respond.

Sincerely,

Mario J. Valladares-Garrido

Universidad Continental, Lima 15046, Peru; mvalladares@continental.edu.pe
