# Supplementary material for: Smartphone dependence, addiction, and insomnia among medical students during the COVID-19 pandemic
Source: PLoS One. 2025 Jul 31;20(7):e0329495. doi: 10.1371/journal.pone.0329495 (PMC12312953; doi:10.1371/journal.pone.0329495)
Supplement: Attachment — Submitted filename: Response_to_Reviewers_auresp_2.pdf [file pone.0329495.s006.pdf]

Dear **Editor**,

Thank you very much for reviewing our article, " ***Smartphone dependence, addiction, and insomnia among medical students during the COVID-19 pandemic*** ". Your suggestions and comments will be addressed below. Thank you for your valuable time and excellent review.

Editor's comments

1. **Editor says:** The authors used both the terms dependence and addiction in the text. Somewhere they are distinct and somewhere they are interchanged. I would suggest to change this to avoid confusion and to use only one term or to clearly explain the difference between then in the text.

   Our response: Thank you for your valuable observation regarding the inconsistent use of the terms dependence and addiction throughout the manuscript. We acknowledge that in the previous version, these terms were sometimes used interchangeably and without sufficient conceptual clarification, which could lead to confusion for readers.
   In response, we have carefully revised the manuscript to ensure precise and consistent terminology aligned with the structure of the Smartphone Dependence and Addiction Scale (SDAS), the instrument used in this study. This scale classifies smartphone use into three categories—nondependence, dependence, and addiction—based on increasing severity. To clarify this, we have added a concise conceptual distinction in the Materials and Methods section, stating that dependence refers to problematic but still functional smartphone use, whereas addiction denotes a more severe level, marked by loss of control and functional impairment. This clarification has also been echoed in the Introduction, the Abstract, and the Discussion to maintain internal consistency. Moreover, in the Results section and tables, we have preserved both categories to respect the original scoring structure of the SDAS, using fixed cutoff values (≤2.23, 2.24–3.05, ≥3.06) established during the original validation study. A corresponding explanatory note has also been added to Table 3 to reflect this, and all prior references to sample-based percentiles have been corrected.

2. **Editor says:** The SDAS cutoff points are pooled from the sample so they are sample dependent. They don't give absolute numbers which would be more sample independent. I would like to see more explanation abut this. With that kind of cutoff points any sample would produce both addictive and non-addictive participants.

   Our response: Thank you for the opportunity to clarify this methodological point. We understand the concern regarding the use of percentile-based cutoffs, which would indeed render the classification of smartphone use levels sample-dependent and limit generalizability. However, we would like to clarify that we did not generate cutoffs from our own sample distribution. Instead, we followed the original scoring criteria proposed by Aranda-López et al. (2017) in the development and validation of the "Escala de Dependencia y Adicción al Smartphone (EDAS)". This validated scale establishes fixed thresholds based on average item scores to classify users into three categories:
   - Nondependent use: mean score ≤ 2.23
   - Dependent use: mean score between 2.24 and 3.05
   - Addictive use: mean score ≥ 3.06

   These thresholds were applied directly in our research and therefore do not vary across samples. The categorization in our study is sample-independent and consistent with the instrument's validated structure. To avoid further confusion, we have revised the Materials and Methods section to explicitly state that the classification was based on predefined, validated cutoff scores from the original scale, and not on percentiles derived from our study sample.

3. **Editor says:** The authors wrote `We found that smartphone dependence increased the prevalence of insomnia by 44.2%.` This result is from chi square test of association, so we can not draw conclusion what is the cause. Maybe it was the other way round or some third factor influencing both of those. The authors should change the language used to describe this result.

   Our response: Thank you for the valuable observation. We have revised the language in the Abstract, Results, and Discussion to ensure that only associations are reported, avoiding any implication of causality. The revised phrasing reflects that smartphone dependence and addiction were associated with higher prevalence of insomnia. We also added a statement in the Discussion and Conclusions acknowledging that the cross-sectional design does not permit causal inference.

4. **Editor says:** The same applies to `depression increased the prevalence of insomnia`. It is test of association.

   Our response: Thank you for the observation. We have revised the language to reflect that depression was associated with a higher prevalence of insomnia, rather than suggesting a causal relationship. This change has been made in the Results and Discussion sections to ensure consistency with the cross-sectional nature of the study.

Reviewer #4:

1. **Reviewer says:** Thank you for the opportunity to review the manuscript entitled "Smartphone dependence, addiction, and insomnia among medical students during the COVID-19 pandemic" (Manuscript ID: PONE-D-24-56765). The manuscript addresses an important and timely topic, particularly given the global reliance on digital devices and the mental health challenges exacerbated by the COVID-19 pandemic.
   Our response: Thank you for your positive feedback and for recognizing the relevance of our study. We appreciate your comments and have addressed them carefully to improve the manuscript.

2. **Reviewer says:** The study is well-structured, the statistical analysis is appropriate, and the discussion is grounded in relevant literature. The use of validated instruments (ISI, PHQ-9, GAD-7, and SDAS) strengthens the methodological rigor, and the findings contribute meaningfully to the existing knowledge on smartphone dependence and sleep health in medical student populations.
   Our response: Thank you for your encouraging remarks. We appreciate your recognition of the study's structure, methodological rigor, and contribution to the literature.

3. **Reviewer says:** The large sample size (n = 370) provides adequate power for the analyses. Adjustments for potential confounders such as depression and anxiety enhance the internal validity of the results.
   Our response: Thank you for highlighting the strengths of our study design. We appreciate your recognition of the sample size and the inclusion of relevant covariates to improve internal validity.

4. **Reviewer says:** The study uses a well-defined and operationalized construct of smartphone dependence and addiction using a validated scale adapted for Spanish-speaking populations.
   Our response: Thank you for acknowledging the use of a validated and culturally adapted scale. We agree that employing a well-defined construct enhances the robustness and relevance of our findings.

5. **Reviewer says:** Clarity on Secondary Analysis:

While the authors mention this is a secondary analysis of previously collected data (ref [21]), greater clarity should be provided regarding the original study's objectives, overlap in variables analyzed, and the novelty of this manuscript's specific focus. This is essential to fully assess the extent of original contribution and avoid concerns of salami publication or dual submission.
Our response: Thank you for this important observation. We confirm that the current manuscript is a secondary analysis of data originally collected for the study published as reference [21] (Santander-Hernández et al., PLOS ONE, 2022). In that study, the primary objective was to assess the association between smartphone overuse and symptoms of depression and anxiety. In contrast, the present manuscript is based on a different research question, focusing on the role of smartphone dependence and addiction—as defined by validated SDAS cutoffs—as the main exposure, and insomnia as the primary outcome. These variables (exposure and outcome) were not analyzed or reported in the original publication. Therefore, the analytic model, outcome of interest, and conceptual framing are all distinct, providing an original contribution to literature. We have now revised the Methods section to clearly state the objective of the primary study, the overlap in instruments, and the novelty of the present focus. We also confirm that there is no duplication or overlap in results, and that this manuscript addresses a unique and independent line of inquiry.

6. **Reviewer says:** Sampling and Generalizability:
The convenience sampling strategy and use of WhatsApp groups may limit generalizability. While this is acknowledged in the discussion, it may be helpful to provide more context or comparison with national student demographics to assess representativeness.
Our response: Thank you for this insightful suggestion. We acknowledge that the use of convenience sampling through WhatsApp groups may limit the generalizability of our findings. However, as noted in the revised Discussion section, recent multicenter mental health studies conducted in Peru among medical students during the COVID-19 pandemic have reported demographic characteristics—particularly in terms of age and sex—similar to those observed in our sample [PMID: 35201957, 35967544, 37923417, 37363900]. This comparability supports the representativeness of our sample and adds contextual value to the interpretation and applicability of our results despite the non-random sampling method.

7. **Reviewer says:** Data Availability:
The manuscript states "All relevant data are within the manuscript and its Supporting Information files." However, it would be helpful to explicitly indicate where the datasets can be accessed or whether any anonymized data files are provided for replication purposes.
Our response: Thank you for this helpful suggestion. We have updated the Data Availability Statement to explicitly clarify that the anonymized dataset used for this analysis is included as a Supporting Information file (S1 Table. Database.), allowing full replication of the study. All relevant variables and coding procedures are documented for transparency and reproducibility.

8. **Reviewer says:** Ethical Considerations: The authors adequately describe IRB approval and informed consent. However, it would strengthen the ethical narrative to specify whether participants were compensated (monetarily or through academic credits) and confirm that participation did not influence course evaluations or academic standing.

   Our response: Thank you for this important observation. We confirm that participants were not offered any monetary compensation or academic credit for their participation. Additionally, we clarify that participation was entirely voluntary and had no influence on course evaluations, grades, or academic standing. This information has been added to the Ethics Statement in the Materials and Methods section to strengthen the ethical transparency of the study.

9. **Reviewer says:** Language and Formatting:
   A few sections would benefit from minor editorial revisions for clarity and flow, particularly in the Methods and Discussion. For instance, "not single" could be more clearly described as "married or in a relationship."

   Our response: Thank you for your helpful observation. We have reviewed the manuscript for clarity and language consistency, particularly in the *Methods* and *Discussion* sections. The phrase "not single" has been revised to "married or in a relationship," and other minor editorial improvements have been made throughout the manuscript to enhance readability and flow.

10. **Reviewer says:** Future Directions:
   While the authors suggest longitudinal and comparative studies in the future, it may be valuable to highlight the potential of interventions (e.g., smartphone use reduction strategies or sleep hygiene programs) that could stem from this evidence.
   Our response: Thank you for this thoughtful recommendation. In response, we have expanded the *Discussion* section to emphasize the practical relevance of our findings. Specifically, we now highlight the potential for developing and evaluating structured interventions—such as digital well-being education, sleep hygiene programs, and cognitive-behavioral strategies—that aim to mitigate insomnia and support mental health among medical students. This addition aligns with the observed associations and reinforces the translational value of our results.

11. **Reviewer says:** Ethics and Integrity:
   There are no apparent concerns related to research misconduct or unethical practices. The ethics approval is clearly stated (CEI-EPM-UCV-2020), and there is no indication of duplicate publication at this time. However, the authors should ensure that the current manuscript provides distinct contributions relative to the prior publication (ref [21]).
   Our response: Our response: Thank you for this observation. We confirm that this manuscript is a secondary analysis of data previously collected for a different research objective, as outlined in reference [21]. The present study addresses a distinct research question by examining the association between smartphone dependence/addiction and insomnia—variables not analyzed or reported in the prior publication. We have clarified this distinction in the Study Design subsection of the Materials and Methods section, ensuring full transparency and reinforcing the originality of the current contribution. There is no duplication of results or overlap in conclusions.

If you have any comments or recommendations, we are ready to respond.

Sincerely,

Mario J. Valladares-Garrido

Escuela de Medicina Humana, Universidad Señor de Sipán, Chiclayo, Peru; vgarrido@uss.edu.pe
